# Postoperative rehospitalization in older surgical patients: an age-stratified analysis

**DOI:** 10.1186/s13741-023-00313-3

**Published:** 2023-06-21

**Authors:** Caroline Andrew, Christina M. Fleischer, Pablo Martinez Camblor, Vinca Chow, Alexandra Briggs, Stacie Deiner

**Affiliations:** 1 Department of Anesthesia, Critical Care, and Pain Medicine, Massachusetts General Hospital, Boston, MA USA; 2 Department of General Surgery, Michigan Medicine, University of Michigan, Ann Arbor, MI USA; 3 Department of Anesthesiology, Dartmouth Hitchcock Medical Center, Lebanon, NH 03755 USA; 4 Department of Surgery, Dartmouth Hitchcock Medical Center, Lebanon, NH 03755 USA

**Keywords:** Geriatrics, Surgery, Rehospitalization, Complications

## Abstract

**Background:**

Older adults comprise 40% of surgical inpatients and are at increased risk of postoperative rehospitalization. A decade ago, 30-day rehospitalizations for Medicare patients were reported as 15%, and more than 70% was attributed to medical causes. In the interim, there have been several large-scale efforts to establish best practice for older patients through surgical quality programs and national initiatives by Medicare and the National Health Service. To understand the current state of rehospitalization in the USA, we sought to report the incidence and cause of 30-day rehospitalization across surgical types by age.

**Study design:**

We performed a retrospective study utilizing the American College of Surgeons National Surgical Quality Improvement Program (ACS NSQIP) dataset from 2015 to 2019. Our primary exposure of interest was age. Patients were categorized into four groups: 18–49, 50–64, 65–74, and 75 + years old. Reasons for rehospitalization were evaluated using NSQIP defined causes and reported International Classification of Disease (ICD)-9 and ICD-10 codes. Our primary outcome was the incidence of unplanned 30-day rehospitalization and secondary outcome the cause for rehospitalization. Variables were summarized by age group through relative (%) and absolute (n) frequencies; chi-square tests were used to compare proportions. Since rehospitalization is a time-to-event outcome in which death is a competing event, the cumulative incidence of rehospitalization at 30 days was estimated using the procedure proposed by Gray. The same strategy was used for estimating the cumulative incidence for unplanned rehospitalizations.

**Results:**

A total of 2,798,486 patients met inclusion criteria; 198,542 had unplanned rehospitalization (overall 7.09%). Rehospitalization by age category was 6.12, 6.99, 7.50, and 9.50% for ages 18–49, 50–64, 65–74, and 75 + , respectively. Complications related to the digestive system were the single most common cause of rehospitalization across age groups. Surgical site infection was the second most common cause, with the relative frequency decreasing with age as follows: 21.74%, 19.08%, 15.09%, and 9.44% (*p* < .0001). Medical causes such as circulatory or respiratory complications were more common with increasing age (2.10%, 4.43%, 6.27%, 8.86% and 3.27, 4.51, 6.07, 8.11%, respectively).

**Conclusion:**

We observed a decrease in overall rehospitalization for older surgical patients compared to studies a decade ago. The oldest (≥ 75) surgical patients had the highest 30-day rehospitalization rates (9.50%). The single most common reason for rehospitalization was the same across age groups and likely attributed to surgery (ileus). However, the aggregate of medical causes of rehospitalization was more common in older patients; surgical and respiratory reasons were twice as common in this group. Rehospitalization increased by age for some surgery types, e.g., lower extremity bypass, more than others, e.g., ventral hernia repair. Future investigations should focus on interventions to reduce medical complications and further decrease postoperative rehospitalization for older surgical patients undergoing high-risk procedures.

**Supplementary Information:**

The online version contains supplementary material available at 10.1186/s13741-023-00313-3.

## Introduction

Adults over 65 years of age represent a significant and growing proportion of surgical patients around the world (Centers for Disease Control 2010; Fowler et al. 2019). Rehospitalization in general is most common in older Medicare patients. Furthermore, at least 25% of rehospitalizations in medical patients have been shown to be preventable (Lee et al. 2017). A decade ago, Jencks et al. published their seminal paper describing the patterns and frequency of readmission in Medicare patients (Jencks et al. 2009). At that time, 15% of all Medicare beneficiaries with a surgical hospitalization were rehospitalized within 30 days of discharge (Jencks et al. 2009). In the interim, there have been some targeted efforts to reduce rehospitalization for many types of surgeries including enhanced recovery protocols and guidance by the American College of Surgeons to improve perioperative care for older adults.

Current data regarding the incidence and cause of hospitalization postoperatively by age category is lacking. Prior work has described causes of rehospitalizations for a single procedure, which is useful but limited with respect to the specific vulnerability of older adults. Given that a proportion of rehospitalizations are preventable, defining which causes are most common may identify areas amenable to intervention. For example, if medical causes predominate, then future study should focus on transitional care; if surgical quality is an issue, then procedure-specific technique requires greater attention.

To address the causes of rehospitalization by age category, we utilized the American College of Surgeons National Surgical Quality Improvement Program (ACS NSQIP) dataset and compared patients aged 18–49, 50–64, 64–74, and 75 and older. The higher incidence of medical comorbidities in older adults predisposes them to complications related to underlying chronic disease (Merkow et al. 2015; Glans et al. 2020; Palmisano et al. 2018; Hines et al. 2011). Therefore, we hypothesize that rehospitalization in older adults continues to be related to medical reasons (e.g., cardiac complications, medication issues) and less frequently due to surgery-related reasons (e.g., surgical site infections and ileus). If management of chronic conditions or primarily medical complications contributes significantly to rehospitalization for older adults, then this will be an avenue for future intervention.

## Methods

This study was reviewed by the Dartmouth Hitchcock Human Research Protection Program and deemed “Not Human Subjects Research” on June 2, 2021. Permission for the ACS NSQIP Participant Use File (PUF) was obtained with permission from our institutional NSQIP official.

### Data source

The American College of Surgeons National Quality Improvement Project is a national database that includes over 700 hospitals across the USA. Participant institutions contribute data gathered from medical charts by trained abstractors. Detailed data including basic demographics, comorbidities, operative interventions, complications, and rehospitalizations are collected through standardized methods. Complete information regarding data variables and collection methods are well described in ACS NSQIP publications and other literature (American College of Surgeons National Surgical Quality Improvement Program 2022; Ingraham et al. 2010; Shiloach et al. 2010; Maggard-Gibbons 2014). The data is available to participating hospitals to improve their quality by understanding their patient outcomes and comparing with other institutions. ACS NSQIP data is also available for research.

All ACS NSQIP procedures with an inpatient stay from 2015 to 2019 were initially considered for the study. Exclusion criteria included the following: obstetrics, rehospitalization related to pregnancy, radiology, length of stay greater than 30 days after the surgical procedure, death prior to discharge, missing rehospitalization status, planned rehospitalizations after surgery, and outpatient procedures with subsequent admissions.

### Patients and predictors

Our exposure of interest was age group. Patients were grouped into four age categories: 18–49, 50–64, 64–74, and 75 years and older. We identified and present confounders of the relationship between age and readmission including sex, comorbidity as represented by the American Society of Anesthesiology Physical Status (ASA), weight, frailty, functional status (dependent), and surgical procedure. To assess the role of surgical procedure and variation in rehospitalization by age category, we reproduced the surgical categories as selected by Merkow et al. for clinical and policy relevance (Merkow et al. 2015). Regarding frailty in the dataset, we used the mFI (modified frailty index), a NSQIP-based factor index, which has been validated to reflect frailty and predict morbidity and mortality at a cutoff of mFI ≥ 2 (Subramaniam et al. 2018).

### Rehospitalization

Unplanned rehospitalization within 30 days was our primary outcome. Rehospitalization in ACS NSQIP was coded as planned versus unplanned and related or unrelated to the index hospitalization. The distinctions were made by the NSQIP participating hospital’s data abstractor. The primary suspected cause of rehospitalization was reported based upon either the standard NSQIP complication categories (using the variable “READMSUSREASON1”) or by ICD9 or ICD10 codes if the cause was not included within the prior categories. The category or ICD code was selected by the data abstractor. For this study, ICD9 and ICD10 codes were grouped in order to analyze causes in clinically relevant categories. We present the most frequent causes of unplanned rehospitalization for each age group including all with a frequency of at least 3% in one of the age groups.

Time to rehospitalization in the NSQIP dataset was reported as days between operative procedure and rehospitalization. To calculate the time from discharge to rehospitalization, the difference between time to rehospitalization and length of stay was calculated. For patients with missing length-of-stay (LOS) data, the median LOS for that procedure was used. For patients with missing time to rehospitalization data, the median time to rehospitalization for that cause was used.

### Statistical analysis

Demographic variables were summarized by age group through relative (%) and absolute (*n*) frequencies; chi-square tests were used to compare proportions. We performed a sensitivity analysis to examine mortality during the index hospitalization and hospitalization greater than 30 days since both were exclusion criteria but also increase with age. The cumulative incidence of rehospitalization at 30 days was estimated using the procedure proposed by Gray because rehospitalization is a time-to-event outcome in which death is a competing event (Gray 1988). The nonparametric Gray estimator allows the estimation of the cumulative incidence function without assuming independence between the censorship and the event. Therefore, this is the adequate procedure to use in competing risk settings. The provided test is similar to the long-rank test in the competing risk context. The same strategy was used for estimating the cumulative incidence for unplanned rehospitalizations, given that planned rehospitalizations would be an additional competing event. The cause of rehospitalization was described by age group with relative (%) and absolute (n) frequencies, using chi-square tests to compare proportions. Regarding missing data, subjects were excluded if readmission status was missing. Missing time to readmission was replaced by the median time of readmission by cause of readmission. The median length of stay by type of surgery replaced missing length of stay. A case-complete approach was used for the rest of variables, i.e., only patients with the variable of interest were included for that particular analysis. All statistical analyses were accomplished utilizing R (www.r-project.org), in specific, package cmprsk and factoextra.

## Results

**Fig. 1:**
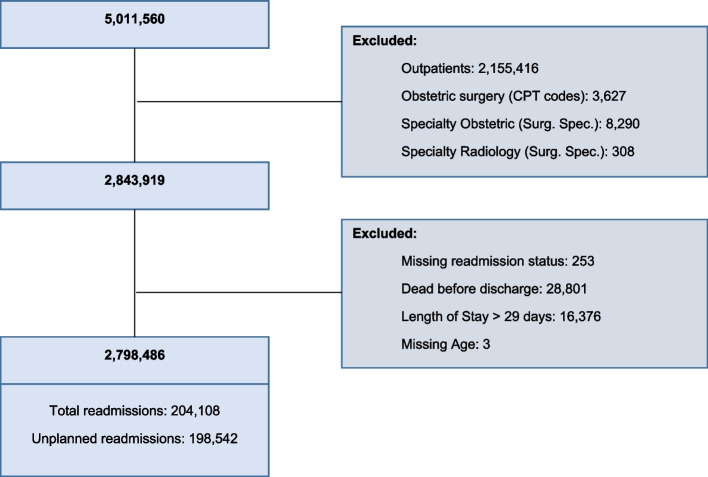
STROBE diagram of the NSQIP cohort

Between 2015 and 2019, a total of 5,011,560 patients underwent surgical procedures. We excluded outpatient procedures (*n* = 2,155,416), patients with obstetric surgery CPT codes (*n* = 3627), procedures where surgical specialty was listed as obstetrics (*n* = 8290), radiology (*n* = 308), patients who died during the index hospitalization (*n* = 28,801), remained as inpatients 30 days after their procedure (n = 16,376), patients with missing rehospitalization status (*n* = 253), or age (3). The final cohort contained 2,798,486 patients (Fig. 1).

### Demographic and comorbidities (Table 1)

**Table 1:** Preoperative patient factors and surgical type

	Age (18–49) *N* = 678,316 (24.24)	Age (50–64) *N* = 888,811 (31.760)	Age (65–74) *N* = 695,795 (24.86)	Age + 75 *N* = 535,564 (19.14)	
	*N* (%)	*N* (%)	*N* (%)	*N* (%)	*p*-value
Gender, male	250,324 (36.90)	417,056 (46.92)	336,217 (48.32)	231,379 (43.20)	< 0.0001
ASA class					< 0.0001
I and II	436,493 (64.35)	384,252 (43.23)	234,442 (33.69)	113,502 (21.19)	
III	219,135 (32.31)	440,303 (49.54)	392,512 (56.41)	336,065 (62.75)	
IV and V	21,093 (3.11)	62,472 (7.03)	67,366 (9.68)	84,711 (15.82)	
None assigned	1595 (0.24)	1784 (0.20)	1475 (0.21)	1286 (0.24)	
Body mass index (BMI)					< 0.0001
Underweight	11,818 (1.74)	13,317 (1.50)	11,015 (1.58)	16,843 (3.14)	
Normal	146,113 (21.54)	165,541 (18.62)	141,408 (20.32)	166,956 (31.17)	
Overweight	167,493 (24.69)	249,704 (28.09)	221,664 (31.86)	183,758 (34.31)	
Obese	329,613 (48.59)	444,258 (49.98)	30,9319 (44.46)	146,670 (27.39)	
Emergency	115,763 (17.07)	78,359 (8.82)	53,575 (7.70)	76,934 (14.37)	< 0.0001
Dependent functional status	9655 (1.42)	20,551 (2.31)	23,267 (3.34)	53,068 (9.91)	
mFI-5 (groups)					< 0.0001
< 2	636,797 (93.88)	715,724 (80.53)	509,093 (73.17)	371,335 (69.34)	
≥ 2	41,518 (6.12)	173,087 (19.47)	186,702 (26.83)	164,229 (30.66)	
Surgical specialty					< 0.0001
Cardiac	1837 (0.27)	6420 (0.72)	6701 (0.96)	4581 (0.86)	
General	387,072 (57.06)	358,946 (40.38)	224,185 (32.22)	164,899 (30.79)	
Gynecology	98,190 (14.48)	47,085 (5.30)	21,735 (3.12)	10,062 (1.88)	
Neurosurgery	48,331 (7.13)	70,753 (7.96)	48,396 (6.96)	25,421 (4.75)	
Orthopedics	72,455 (10.68)	247,918 (27.89)	246,532 (35.43)	225,159 (42.04)	
Otolaryngology (ENT)	14,545 (2.14)	12,295 (1.38)	7031 (1.01)	4215 (0.79)	
Plastics	16,774 (2.47)	13,696 (1.54)	4315 (0.62)	1637 (0.31)	
Thoracic	8310 (1.23)	18,019 (2.03)	18,026 (2.59)	9867 (1.84)	
Urology	18,466 (2.72)	57,417 (6.46)	50,716 (7.29)	27,396 (5.12)	
Vascular	12,335 (1.82)	56,262 (6.33)	68,158 (9.80)	62,327 (11.64)	

Number of surgical patients overall was equally distributed across age groups: 24.24, 31.76, 24.86, and 19.15%. The oldest patients were more often underweight (3.14%), physically frail (defined as an mFI score ≥ 2, 30.66%), or had a dependent functional status (9.91%) prior to surgery. For the patients age 18–49, 1.74% were underweight, 6.12% had an mFI score ≥ 2, and 1.42% had a dependent functional status. A greater proportion of older patients were ASA IV/V (condition that is a threat to life or not expected to survive) 3.11, 7.03, 9.68%, and 15.82% for the 18–49, 50–64, 65–74, and 75 years and older groups, respectively. Emergency, surgery was most common in the youngest group 17.07% and the increased with age 8.82, 7.70, and 14.37. An extended list of comorbidities by age condition is available in the supplementary material.

### Unplanned rehospitalization (Table 2)

**Table 2:** Unplanned postoperative rehospitalizations for select surgical subtypes

	**Overall**	**Bariatric**	**Lower extremity vascular bypass**	**Colectomy or proctectomy**	**Ventral hernia repair**	**Hysterectomy**	**Total hip or knee arthroplasty**	**Others**
	*N* (%)	*N* (%)	*N* (%)	*N* (%)	*N* (%)	*N* (%)	*N* (%)	*N* (%)
Total	198,542 (7.36)	4595 (4.16)	5228 (15.82)	33,094 (11.25)	6685 (8.75)	6577 (4.51)	18,707 (3.73)	123,656 (8.06)
Age 18–49	40,501 (6.12)	2850 (4.01)	257 (16.28)	7105 (11.88))	1652 (8.17)	3380 (4.20)	850 (3.31)	24,407 (6.06)
Age 50–64	60,026 (6.99)	1370 (4.24)	1809 (15.70)	10,577 (10.33)	2579 (8.73)	1832 (4.49)	5079 (2.85)	36,780 (7.93)
Age 65–74	50,160 (7.50)	311 (5.04)	1692 (14.50)	8107 (11.04)	1543 (9.06)	925 (5.40)	6040 (3.34)	31,542 (8.71)
Age + 75	47,855 (9.50)	64 (7.66)	1470 (17.68)	7305 (12.47)	911 (9.69)	440 (6.10)	6738 (5.78)	30,927 (10.22)

**Fig. 2:**
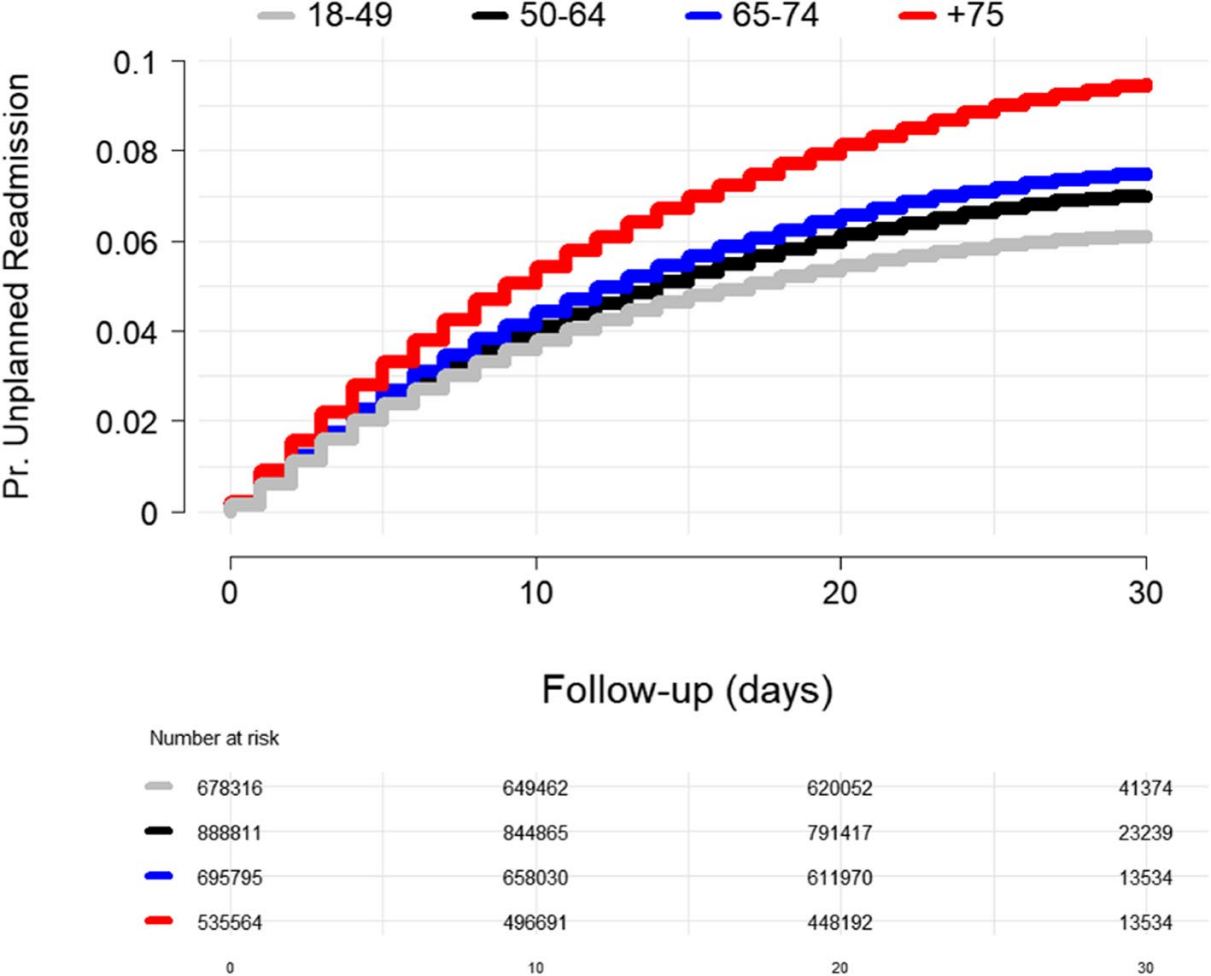
Proportion of patients rehospitalized by age category over 30-day follow-up

The cumulative incidence of 30-day unplanned rehospitalization increased with age, 6.12%, 6.99%, 7.50%, and 9.50%. The proportion of unplanned rehospitalization increased over 30 days; the increase was most notable in the 75 and older group (Fig. 2). The proportion of unplanned rehospitalization differed by surgical type, although none by more than 3 percentage points. However, the incidence of rehospitalization did not always increase with age; each surgical type had a distinct pattern. In lower extremity vascular bypass patients, 17.68% of patients 75 years were rehospitalized within 30 days, but the second highest incidence of rehospitalization was in the youngest group 18–64, 16.28%. Total hip or knee arthroplasty had fairly similar rehospitalization by age except for the oldest group: 3.31% rehospitalization rate for the 18–49, 2.85% for 50–64, 3.34% for 65–74, and 5.78% for the 75 years and older group. Ventral hernia repair was similar across age categories: 8.17, 8.73, 9.06, and 9.69% for the 18–49, 50–64, 65–74, and 75 and older group.

### Causes of unplanned rehospitalizations (Table 3)

**Table 3:** Most common reasons^a^ for unplanned rehospitalizations

	Age (18–49)	Age (50–64)	Age (65–74)	Age + 75	
	*N* (%)	*N* (%)	*N* (%)	*N* (%)	*p*-value
Symptoms/disease of the digestive system	10,667 (26.34)	12,109 (20.17)	8964 (17.87)	7653 (15.99)	< 0.0001
Surgical site infection	8801 (21.73)	11,454 (19.08)	7572 (15.09)	4520 (9.44)	< 0.0001
#Injury/poisoning/external causes	2384 (5.89)	4226 (7.04)	3507 (6.99)	3464 (7.24)	< 0.0001
Symptoms/disease of the respiratory system	1326 (3.27)	2710 (4.51)	3043 (6.07)	3883 (8.11)	< 0.0001
Endocrine/nutritional/metabolic	1508 (3.72)	2214 (3.69)	1834 (3.66)	1735 (3.63)	0.8791
Symptoms/disease of the circulatory system	851 (2.10)	2662 (4.43)	3147 (6.27)	4240 (8.86)	< 0.0001
Symptoms/disease of the nervous system	1560 (3.85)	1650 (2.75)	1254 (2.50)	979 (2.05)	< 0.0001
Sepsis	1167 (2.88)	2017 (3.36)	1933 (3.85)	1904 (3.98)	< 0.0001
$General symptoms and signs	995 (2.46)	1537 (2.56)	1408 (2.81)	1569 (3.28)	< 0.0001
Urinary tract infection	494 (1.22)	982 (1.64)	1170 (2.33)	1601 (3.35)	< 0.0001

^a^Frequency of 3% or larger. $General symptoms and signs include codes ICD9 780 and ICD10 R50-69. #Injury/poisoning/external causes include falls with injury: codes ICD9 800–999 and ICD10 S00-T88

The most common cause of rehospitalization across all age groups was symptoms/disease of the digestive system: 26.34%, 20.17%, 17.87%, and 15.99%, for the 18–49, 50–64, 65–74, and 75 years and older groups, respectively (*p* < 0.0001). Surgical site infection (SSI) was the second most common cause across all age groups; however, the relative frequency decreased significantly with increasing age: 21.73, 19.08, 15.09, and 9.44% for the 18–49, 50–64, 65–74, and 75 years and older groups, respectively (*p* < 0.0001). For the 75 years and older group, the next most common causes of rehospitalization were as follows: symptoms/disease of the circulatory system (8.86%) and symptoms/disease of the respiratory system (8.11%). For the 18–49- and 50–64-year-old group, injury/poisoning/external causes were the third most common cause of rehospitalization (5.89% and 7.04%, respectively). Urinary tract infections represented 1.22% of rehospitalizations in the 18–49 year old group, 1.64% of the 50–64-year-old group, 2.33% of the 65–74-year-old group, and 3.35% of the 75 years and older group.

## Discussion

We conducted a retrospective analysis utilizing ACS NSQIP data from 2015 to 2019 comparing the incidence and reason for unplanned postoperative rehospitalizations across four age groups. We found that the incidence of 30-day unplanned rehospitalizations increases with age. The most common reason for rehospitalization was complications of the GI tract. The second most common reason was surgical site infection which decreased with increasing age. For the 75 years and older group, the third and fourth most common causes of rehospitalization were symptoms/disease of the circulatory system (8.86%) and respiratory system (8.11%), which together accounted for more rehospitalizations than surgical site infections alone in this age group (9.44%). By surgical procedure type, some procedure types had a greater readmission by age, but not all. This suggests that there is not a “one-size-fits-all” solution to prevent rehospitalization, but rather, each specialty needs to be aware of the role of age when considering 30-day outcomes.

Compared to Jencks et al., who published in 2009 utilizing 2003–2004 Medicare data, our overall incidence of rehospitalization was lower (15.6% vs. 9.50%) (Jencks et al. 2009). However, our incidence was similar to Merkow et al. who reported a single year of NSQIP data from 2012 (Merkow et al. 2015). Compared to surgical patients in the UK, our overall rehospitalization rate is higher. Lee et al. described a 30-day rehospitalization of 4.6% in 2017 for general surgery patients (Lee et al. 2017). Our overall rehospitalization for ventral hernias (a somewhat less invasive general surgery procedure) was on average is 8.75%, and our incidence for colectomy or proctocolectomy (more invasive general surgery procedure) is 11.5%. Lee et al. found that in the National Health System and within the group of general surgery patients, patient variables were not associated with rehospitalization, and 40% were potentially avoidable (Lee et al. 2017). Regarding the causes of rehospitalization, Jencks et al. commented that nearly 70% of the 30-day surgical rehospitalization in their cohort was attributed to medical causes (Jencks et al. 2009). Currently, there is no standard variable in NSQIP which categorizes rehospitalization as “medical” vs. “surgical.” We did note that in our population, the sum of surgical site infections and symptoms/diseases of the digestive system (including ileus, considered secondary to surgery and pain medications) accounted for 30% and 25% of the 65–74 and 75 years and older rehospitalizations, a significant proportion of medical rehospitalizations.

When stratifying by both age and surgery type, we observed that the degree of variation across age groups was not uniform across surgery types. For example, there was a notable difference in rate of rehospitalization across age cohorts for lower extremity vascular bypass and total hip or knee procedures, while the incidence of rehospitalizations was much more consistent across all age groups for ventral hernia repair. This observation suggests that there may be an underlying association with procedure type and/or the patient population undergoing specific procedures, highlighting important areas for future study and intervention. Age-group targeted interventions for reducing rehospitalizations may be most impactful in the surgical subspecialties with a larger between-group difference.

Limitations include those of a retrospective analysis; we report associations and not causality. Many of our findings are reported using “broad strokes” surgical categories such as “bariatric.” Future studies may seek to further refine our findings for procedures within a category in order to develop protocols to prevent rehospitalization. Similarly, causes of rehospitalization categories are broad, and further identification of the major causes within each diagnosis category is indicated to design interventions. Social determinants of health are extremely important predictors of health outcomes. We were not able to comment on these because NSQIP is entirely de-identified and does not contain any proxy of socioeconomic status such as insurance type or social support. Our data did not include the years during the COVID pandemic. These years are difficult to compare to earlier times as many hospitals decreased the amount and types of elective surgery. Our findings are likely impacted by the fact that the probability of death after surgery and prior to discharge is greater in the oldest group than in the younger group. One could thus assume that the older patients who survived to be readmitted were relatively healthier at baseline, yet still had greater frequency of cardiovascular and respiratory complications than younger patients.

Regarding future directions, the continued relative prevalence of medical causes of rehospitalization for older surgical patients suggests that more formal postoperative transitional medical care may be appropriate. The much lower incidence of rehospitalization in the UK underscores differences in our medical systems. While some of these may be inherent to their system and population, there may be some aspects of the UK workflows which translate to the US system. Since the COVID-19 pandemic, hospitals are under significant pressure to discharge patients earlier, whether this will increase rehospitalization for older surgical patients with complicated medical histories is unclear.

The Jencks study demonstrated that over half of the patients who were rehospitalized within 30 days after discharge did not have an associated claim for an outpatient visit, suggesting that these patients were not seen by a primary care provider between discharge and rehospitalization (Jencks et al. 2009). For medical patients, early primary care after an inpatient hospitalization has shown promising effects on rehospitalization rates (Hernandez et al. 2010; Brooke et al. 2014; Jackson et al. 2015). Additionally, primary care follow-up within 14 days post-discharge has been associated with significantly lower rehospitalization rates among medical patients with high clinical complexity (> 3 chronic conditions) and those undergoing high-risk surgery (Brooke et al. 2014; Jackson et al. 2015). However, several studies have demonstrated that approximately 50% of medical patients do not receive outpatient follow-up in the first 30 days after discharge (Jencks et al. 2009; Jackson et al. 2015; Gilmer and Hamblin 2010). Given the salutary effect of post-discharge follow-up with primary care for medical patients, a logical next step may be to investigate primary care follow-up for surgical patients. A formal approach to post-discharge care is the transitional care model (TCM), which uses advanced practice registered nurses (APRN) to assist patients and their families with the transition between the hospital and the community. These programs have shown promising results in terms of reducing hospital rehospitalization for older patients (> 65^)^ (Naylor et al. 1999; Rich et al. 1995; Morkisch et al. 2020). For example, Naylor et al. studied older adults admitted for common medical and surgical reasons and found that TCMs reduced hospital rehospitalizations while also decreasing the overall cost of care (Naylor et al. 1999). Further research investigating the use of TCMs for postoperative geriatric patients would be beneficial to assess the causality between this multidisciplinary intervention and postoperative rehospitalization rates.

## Conclusions

Our study of patients from US NSQIP participating institutions found that while the incidence of rehospitalization after surgical admission is lower than it was a decade ago, a significant proportion can still be attributed to medical causes. While the single most common causes of rehospitalization was GI related, surgical causes (ileus, wound infection) of rehospitalization became less common with increasing age, while medical causes of rehospitalization increased. Specifically, cardiac and respiratory causes were more common in the 75 years and older group, which highlights the importance of follow-up care for patients with preoperative cardiac and respiratory issues. We found that the degree of variation between age groups was not uniform across surgery types, with certain surgical subtypes, like lower extremity vascular bypass, having a proportionally greater increase in rehospitalizations for the oldest patients. Future studies should consider the implementation of postoperative primary care engagement for older patients undergoing procedures that are high risk for rehospitalization.

## Supplementary Information

**Additional file 1: Supplementary table.** Comorbid conditions by age group.

## Data Availability

Permission for the American College of Surgeons National Surgical Quality Improvement Program (ACS NSQIP) Participant Use File (PUF) was obtained with permission from our institutional NSQIP official. Link to dataset is as follows: ACS NSQIP Participant Use Data File | ACS (facs.org).

## Abbreviations

ACS NSQIP: American College of Surgeons National Surgical Quality Improvement Program
APRN: Advanced registered nurse practitioners
ICD: International Classification of Disease
LOS: Length of stay
mFI: Modified frailty index
PUF: Participant Use File
SSI: Surgical site infection
