# Supplementary material for: Postoperative rehospitalization in older surgical patients: an age-stratified analysis
Source: Perioper Med (Lond). 2023 Jun 21;12:28. doi: 10.1186/s13741-023-00313-3 (PMC10286398; doi:10.1186/s13741-023-00313-3)
Supplement: Supplementary file 1 — Additional file 1: Supplementary table. Comorbid conditions by age group. [file 13741_2023_313_MOESM1_ESM.docx]

Supplementary Table: Comorbid conditions by age group

|  | Age (18-49) | Age (50-64) | Age (65-74) | Age +75 |  |
| --- | --- | --- | --- | --- | --- |
|  | N (%) | N (%) | N (%) | N (%) | p-value |
| Congestive Heart Failure (CHF) | 2108 (0.31) | 7876 (0.89) | 9083 (1.31) | 14096 (2.63) | <0.0001 |
| Chronic Obstructive Pulmonary Disease (COPD) | 5470 (0.81) | 44782 (5.04) | 52616 (7.56) | 46914 (8.76) | <0.0001 |
| Pneumonia | 5258 (0.78) | 11506 (1.29) | 11575 (1.66) | 14512 (2.71) | <0.0001 |
| Diabetes | 59618 (8.79) | 174438 (19.63) | 165210 (23.74) | 110366 (20.61) | <0.0001 |
| Disseminated Cancer | 15600 (2.30) | 36576 (4.12) | 27278 (3.92) | 17167 (3.21) | <0.0001 |
| Dyspnea | 21536 (3.17) | 53632 (6.03) | 57130 (8.21) | 51746 (9.66) | <0.0001 |
| Hypertension | 129859 (19.14) | 445759 (50.15) | 457518 (65.75) | 393956 (73.56) | <0.0001 |
| Bleeding disorder | 12610 (1.86) | 38162 (4.29) | 43009 (6.18) | 55076 (10.28) | <0.0001 |
| Wound Infection | 18576 (1.74) | 34086 (3.84) | 24867 (3.57) | 24156 (4.51) | <0.0001 |
| Renal Failure | 2049 (0.30) | 3999 (0.45) | 3196 (0.46) | 2884 (0.54) | <0.0001 |
| Ventilator dependent | 1955 (0.29) | 30064 (0.34) | 2123 (0.31) | 1459 (0.27) | <0.0001 |
| SIRS or sepsis or septic shock | 69754 (10.28) | 59621 (6.71) | 39033 (5.61) | 42497 (7.93) | <0.0001 |
| Smoker | 143427 (21.14) | 204100 (22.96) | 100047 (14.38) | 35197 (6.57) | <0.0001 |
| Steroid use | 29638 (4.37) | 40312 (4.54) | 33459 (4.81) | 23955 (4.47) | <0.0001 |
| Weight loss>10% | 8845 (1.30) | 16403 (1.85) | 13293 (1.91) | 11686 (2.18) | <0.0001 |
